# A Rare Case of Congenital Factor XIII Deficiency Secondary to Hyperconsumption: A Case Report

**DOI:** 10.7759/cureus.72910

**Published:** 2024-11-03

**Authors:** Amukthamalyada Koduri, Jagadeswar Kakumani, Magesh Kumar S

**Affiliations:** 1 General Medicine, Saveetha Medical College, Chennai, IND

**Keywords:** alcohol abuse, cryoprecipitate, factor xiii, hyperconsumption, rare

## Abstract

Acquired Factor XIII (FXIII) deficiency is a rare condition often associated with underlying medical conditions or medications. We present a case of a 23-year-old male, who presented with prolonged bleeding from a traumatic ulcer site on his left leg. Initial laboratory investigations revealed a severe deficiency in FXIII activity (30%) and antigen levels (25%), with no evidence of congenital disorders or other underlying pathologies. Further evaluation indicated a pattern of hyperconsumption due to ongoing bleeding episodes in the form of excessive use of clotting factors due to repeated bleeding episodes and impaired clot stability, leading to a coagulopathy where factors are consumed faster than they can be produced.

The patient was treated with six units of cryoprecipitate to address the bleeding and was started on high-end antibiotics, including meropenem and vancomycin, to manage a suspected septic process. This comprehensive approach led to significant clinical improvement, with the resolution of symptoms and stabilization of the ulcer site.

This case highlights the importance of considering acquired FXIII deficiency in patients with unexplained bleeding diathesis, especially in the context of alcohol abuse or conditions associated with hyperconsumption. Early recognition and immediate treatment are crucial for improving prognostic outcomes for these patients.

## Introduction

Factor XIII is a critical component of the coagulation cascade, playing a pivotal role in the stabilization of blood clots. It is composed of two subunits, FXIIIA and FXIIIB. FXIIIA is responsible for the enzymatic activity, while FXIIIB serves as a carrier protein, stabilizing the enzyme and enhancing its activity. The primary function of FXIII is to catalyze the cross-linking of fibrin molecules, thereby increasing the density and stability of the blood clot. This cross-linking mechanism is essential for the clot to resist fibrinolysis and maintain its structural integrity during the healing process [1]. 

Deficiency of FXIII is an exceedingly rare bleeding disorder, with an estimated incidence ranging from one in a million to one in five million individuals [2]. It is inherited in an autosomal recessive pattern, meaning both copies of the gene must be defective for the condition to manifest. While congenital FXIII deficiency is well-documented, acquired FXIII deficiency is less common and can occur due to various mechanisms, including hyperconsumption, hypo-synthesis, or an immune-mediated process [3].

In this article, we explored a case of FXIII deficiency, the underlying pathophysiology, clinical presentations, diagnostic challenges, and therapeutic strategies. We also highlighted the importance of a multidisciplinary approach to managing this complex condition, ultimately advocating for increased awareness and research to improve patient outcomes.

## Case presentation

**Figure 1:**
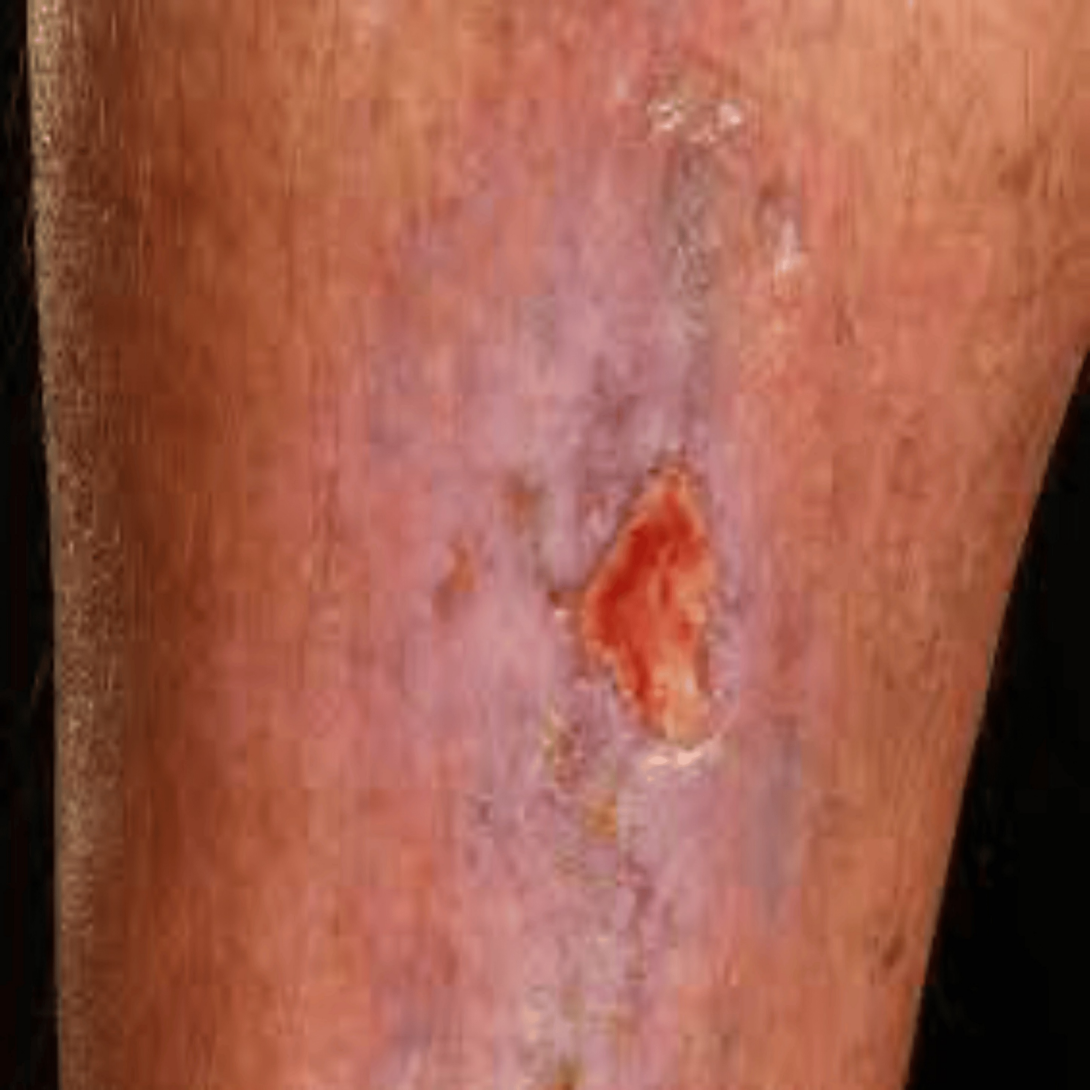
Ulcer site from the wound

A 23-year-old male presented to the Emergency Room (ER) with persistent bleeding from a traumatic ulcer site on his left leg, ongoing for one week. The patient initially sustained trauma to the left leg, which was followed by debridement and initial treatment at an outside facility. Despite these measures, bleeding continued and was unresponsive to conventional treatments. In a span of one week, the wound evolved into an ulcer (Figure 1), and the patient developed a high-grade fever, prompting his visit to the ER.

Upon presentation, the patient’s vital signs were stable, and he had no additional complaints. A comprehensive general and systemic examination revealed localized findings around the ulcer site, including bleeding, clot formation, warmth, tenderness, and surrounding edema. There was no evidence of pedal edema or generalized lymphadenopathy.

The patient's medical history was significant for head trauma sustained at the age of one, which required craniotomy and evacuation of an extradural hemorrhage and posterior fossa hematoma. At that time, he was diagnosed with Factor XIII deficiency based on laboratory tests showing decreased Factor XIII activity (30%) and antigen levels (25%). Genetic testing confirmed the diagnosis by identifying a mutation in the F13A1 gene.

Since the initial diagnosis, the patient had not been on any medications or received regular treatment for his condition.

Laboratory investigations revealed the following (Table 1).

**Table 1:** Laboratory investigative findings of the patient

Parameters	Patient Values	Reference Range
Hemoglobin	9.1 g/dL	13.5-17.5 g/dL
Total leukocyte count (TLC)	17,520 cells/μL	4,000-10,000 cells/μL
International normalized ratio (INR)	1.6	0.8-1.2
Factor XIII activity	30%	70-130%
Factor XIII antigen	25%	70-130%

The elevated international normalized ratio (INR) suggested a coagulation disturbance, prompting the initiation of high-end antibiotics to address a suspected septic process. The patient was started on intravenous meropenem and vancomycin. Given the patient's weight of 68 kg, he was administered six units of cryoprecipitate to manage the bleeding, following the standard protocol of one unit per 10 kg of body weight. The transfusion successfully halted the bleeding, and subsequent INR measurements returned to normal levels.

The wound was treated with daily dressings and continued antibiotics (meropenem and vancomycin). The patient showed significant improvement, with the resolution of symptoms and stabilization of the wound site. Follow-up assessments indicated that the wound was healing appropriately, and plans for outpatient follow-up were made for continued monitoring of his Factor XIII levels and overall health.

## Discussion

Factor XIII deficiency presents a unique challenge in the realm of bleeding disorders. Patients with FXIII deficiency often exhibit normal results in standard coagulation tests, including prothrombin time (PT), activated partial thromboplastin time (aPTT), and platelet counts. This normal appearance in routine tests can obscure the diagnosis, making it imperative to consider FXIII deficiency in cases of unexplained bleeding with normal coagulation profiles. Acquired deficiencies may arise from conditions that consume or destroy FXIII, such as disseminated intravascular coagulation (DIC), severe liver disease, or the presence of inhibitory antibodies [4]. 

In the case of our patient, the history of trauma combined with the prior diagnosis of FXIII deficiency guided the diagnostic process. Despite his normal initial coagulation tests, his bleeding from the ulcer site continuously confirmed the underlying cause of FXIII deficiency, especially given his clinical history. The diagnosis of FXIII deficiency can be complicated due to the normal results of standard coagulation tests. It is crucial for clinicians to maintain a high index of suspicion, particularly in patients presenting with unexplained bleeding [5].

In Factor XIII deficiency, the body attempts to form a stable clot in response to bleeding or tissue injury, but the lack of adequate fibrin cross-linking results in an unstable initial clot. Recurrent bleeding leads to the increased consumption of various clotting factors as the body struggles to stabilize the clot, creating a situation known as consumption coagulopathy where clotting factors are depleted faster than they can be produced. In response to ongoing bleeding, the body attempts to compensate by increasing the production of other coagulation factors, complicating the clinical picture and exacerbating the bleeding tendency.

In acute situations, careful monitoring of the patient's response to treatment is essential. Follow-up assessments of coagulation parameters, including FXIII levels and INR, should guide further management. If bleeding persists despite adequate FXIII replacement, additional investigations may be warranted to identify the potential underlying issues, such as the development of inhibitors against FXIII or other coagulopathy- related conditions [6]. Hence, prompt recognition and effective use of cryoprecipitate or FXIII concentrates can make a significant difference in the management of acute bleeding episodes in patients with FXIII deficiency, highlighting the importance of having these therapeutic options readily available in clinical settings.

In patients with acquired FXIII deficiency, treatment is often directed at the underlying cause. For example, in cases related to autoimmune disorders, immunosuppressive therapy may be indicated. Managing any malignancies or other conditions contributing to the deficiency is essential to resolve the bleeding tendency [7]. A multidisciplinary approach involving hematologists, oncologists, and immunologists is often necessary for comprehensive care.

## Conclusions

Despite its rarity, FXIII deficiency should be considered in patients presenting with severe bleeding and normal routine coagulation test results. A thorough evaluation, including detailed patient history and specialized diagnostic tests, is essential for accurate diagnosis and effective management. Educating patients about their condition is crucial for preventing future complications and ensuring timely intervention.

Early recognition and treatment with appropriate therapies, such as cryoprecipitate or recombinant FXIII, are vital for controlling bleeding and promoting recovery. This case underscores the importance of considering FXIII deficiency in the differential diagnosis of unexplained bleeding and the need for specialized diagnostic and treatment approaches.

The case presented illustrates the complexity of managing FXIII deficiency, highlighting the need for a multidisciplinary approach that includes hematologists, surgeons, and infectious disease specialists. Ongoing education and research are vital in advancing the care for individuals with FXIII deficiency, ultimately leading to improved quality of life and outcomes for affected patients.
